# Metagenomic Insights Into Microbial Diversity of Tea Rhizosphere of the Kangra Valley

**DOI:** 10.1002/mbo3.70416

**Published:** 2026-09-09

**Authors:** Rishu Thakur, Hena Dhar, Shashi Kiran, Arvind Gulati

**Affiliations:** ^1^ CSIR‐Institute of Himalayan Bioresource Technology Palampur Himachal Pradesh India; ^2^ Menzies School of Health Research Charles Darwin University Alice Springs Northern Territory Australia; ^3^ School of Biotechnology, Faculty of Applied Sciences and Biotechnology Shoolini University Solan Himachal Pradesh India; ^4^ Université Paris Cité, INSERM UMR‐S1451, CNRS UMR‐S10253, Institut Necker Enfants Malades Paris France

**Keywords:** amplicon sequencing, Microbiome, soil parameters, tea rhizosphere

## Abstract

This study provides the first metagenomic assessment of microbial diversity from the tea rhizosphere of the Kangra valley. Tea rhizosphere soil samples were collected from 4 locations (Dharamshala, Baijnath, Palampur, and Joginder Nagar) of the Kangra valley. DNA extracts of rhizosphere samples were analysed for bacterial and Archaeal diversity using amplicon sequencing (V3‐V4) region of the 16S rRNA gene and Fungal diversity using ITS1 and ITS2 regions. Baijnath and Palampur samples showed the highest bacterial richness, while Dharamshala and Palampur had the highest fungal richness. Proteobacteria was a dominant phylum in all the rhizosphere samples, followed by Firmicutes, Actinobacteria, Acidobacteria, and Bacteroidetes. A total of 11 fungal phyla were identified among all the locations, with abundance of Ascomycota and Basidiomycota. For the Archaea domain, uncultured archaeon and *Aeropyrum camini* were the most common found among all the locations. A small fraction (< 0.5%) of *Bacillus* and *Pseudomonas* species were observed among all the locations. Alpha and beta diversity indices displayed notable differences within and between microbial diversities. Soil factors were variably associated with microbial diversity, with nitrogen positively aligned with fungal diversity, while EC and K were associated with Archaeal diversity. Soil pH and OM% showed moderate associations with bacterial diversity. These findings provided valuable and comprehensive insights into tea rhizosphere microbial ecology and could be used to better understand microbial functions and their role in plant health.

## Graphical abstract

Tea rhizosphere microbial communities in Kangra Valley.

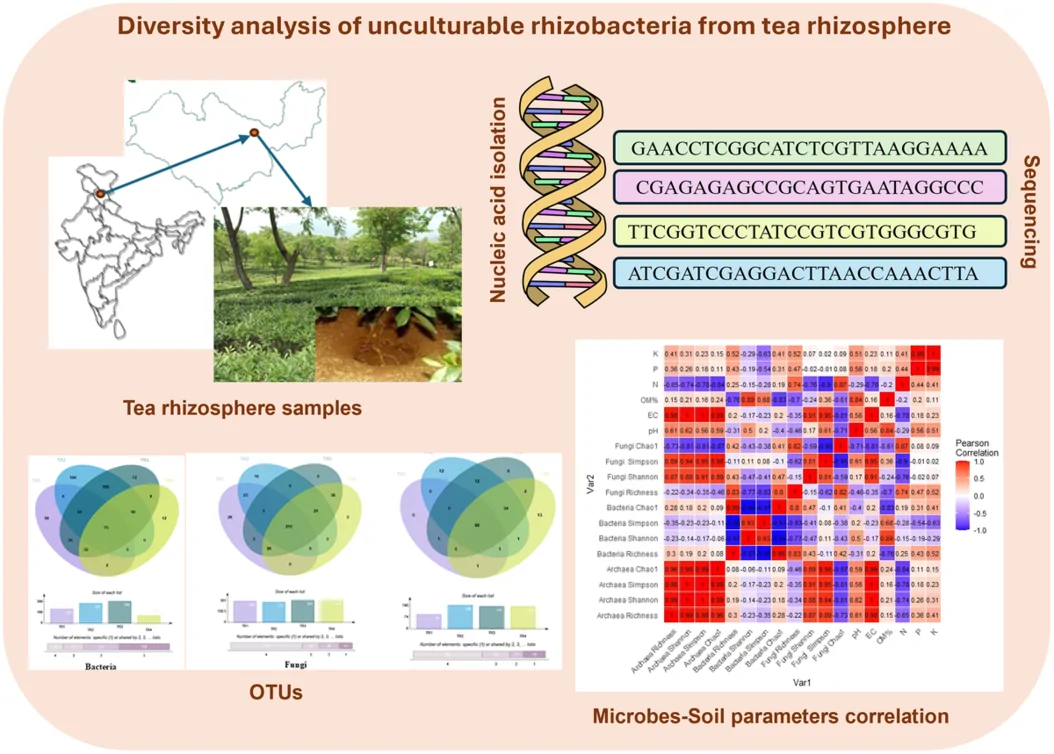

## 1 Introduction

Rhizosphere is a complex and dynamic habitat comprising a diverse microbial composition (Ling et al. 2022). Rhizosphere microbial communities are essential for plant nutrient uptake, growth promotion, stress resilience, and soil health (Luo et al. 2025; Mulugeta et al. 2025). Interactions between plants and microbes are the key determinant of influencing the structural framework and functional processes of plants. Environmental stressors such as flooding, heavy metals, salinity and heavy metal ions concentration significantly impact the microbial compositions. Symbiotic associations between plants and microbial communities enable them to thrive under stressed environmental conditions such as drought, extreme heat/cold, flooding salinity, and metal ion concentration. Several rhizobacteria from the genus including *Acinetobacter*, *Agrobacterium*, *Arthrobacter*, *Bacillus*, *Burkholderia*, *Cellulomonas*, *Enterobacter*, *Halotalea*, *Lysinibacillus*, *Microbacterium*, *Neomicrococcus*, *Paenibacillus*, *Priestia*, *Pseudomonas*, and *Rhizobium* have been reported to enhance plant stress resilience under harsh conditions (Chu et al. 2019; Gulati et al. 2024; Gulati et al. 2024b; Thakur et al. 2024b; Thakur et al. 2025).

Soil physicochemical properties such as soil pH, electric conductivity (EC), organic matter (OM), macronutrients (available N, P, K), micronutrients (Cu, Zn, Fe), and humidity influence the diversity and composition of microbes in the rhizosphere (Xu et al. 2024). Use of fertilizers, pesticides and other amendments to enhance plant growth can also impact the microbial growth (Pantelides et al. 2023). Additionally, climate change impacts such as extreme heat, bushfires, and flash floods are reported to affect the microbial compositions (Tiedje et al. 2022). To fully understand how these factors affect microbial composition, it is important to elucidate microbial diversity. Metagenomic analysis of microbial diversity enables a more comprehensive understanding of soil health and ecosystem functioning.

Tea (*Camellia sinensis*) is a globally important cash crop whose productivity and quality are strongly influenced by soil conditions. Tea is essentially grown in acidic soils, which are generally poor in nutrient availability, making nutrient cycling and microbial activity critical for plant growth. The rhizosphere microbial community plays a key role in supporting tea plant health by regulating nutrient availability, enhancing stress tolerance, and suppressing soil‐borne pathogens. Despite this, the structure and drivers of tea rhizosphere microbial communities remain poorly understood in many agroecological regions. The tea rhizosphere is an excellent example of succession of microbes over several years, as tea plants are perennials and most of the tea plantations have been growing for more than 50 years (Pandey and Palni 1996).

Tea, being a perennial crop, be a perfect spot to explore microbial communities (Jibola‐Shittu et al. 2024). For instance, long‐ standing tea plant age, poor soil nutrients, nitrogen fertilization, chemical or organic fertilizers are sensitive to changes in microbial populations. A study conducted in China reported that continuous tea cultivation altered the soil microbial community with a reduction in the relative abundance of some beneficial bacteria, such as *Bradyrhizobium*, *Mycobacterium*, *Pseudomonas*, *Rhodanobacter*, *and Sphingomonas* (Sun et al. 2022). Long‐term tea monoculture adversely affects soil microbial diversity (Gui et al. 2022). Degradation of soil due to long‐term monoculture of tea plantations is one of the critical issues in tea cultivation. Also, it is still not well explored how different tea varieties and soil properties affect the structure and functions in rhizosphere microbial communities. Microbial diversity analysis, including alpha and beta diversity, of tea rhizosphere can help in understanding the functions of microbes and in harnessing them for better tea production. Assessing alpha and beta diversity in the tea rhizosphere provides insights into microbial community structure and variation, which can help elucidate their potential functions and support strategies for improving tea production.

Previous studies have characterised tea rhizosphere microbial communities in other tea‐growing regions, including Assam and Darjeeling in India and several regions of China, using culture‐independent sequencing approaches. For example, studies in Darjeeling identified Proteobacteria, Actinobacteria, and Acidobacteria as predominant bacterial phyla and demonstrated significant differences in microbial diversity between rhizosphere and bulk soils, while research in Assam using metagenomic sequencing reported *Bacillus*, *Burkholderia*, *Candidatus Solibacter*, *Pseudomonas,* and *Streptomyces* among the dominant rhizosphere bacteria (Bhattacharyya et al. 2022). Studies from China have also demonstrated that tea rhizosphere bacterial communities vary with geographical location, elevation, soil properties and tea variety (Xu et al. 2023). In contrast, the present study provides region‐specific insights into microbial diversity in this important Himalayan tea‐growing region. In our previous paper, we have analysed the microbial and functional diversity of culturable rhizobacteria from the tea rhizosphere of the Kangra valley (Thakur et al. 2025). However, the information on unculturable microbial groups is lacking. A comprehensive understanding of both culturable and unculturable microbial communities is needed to comprehend the dynamics of plant‐microbe interactions. Thus, this work aims to analyse the microbial diversity of unculturable bacteria from the tea rhizosphere and explore the influence of soil physicochemical properties on the composition of these microbes.

## 2 Methods

### 2.1 Study Site and Sample Collection

Tea rhizosphere soil samples were collected from 4 major locations, including Baijnath, Dharamshala, Joginder Nagar, and Palampur (Supporting Information S1: Figure S1), covering the major tea‐growing regions in the Kangra valley. Soil samples (*n* = 16) were collected from a depth of 15–20 cm across 4 locations. Samples collected from the same location were pooled to obtain 4 composite final samples, each representing one sampling site. The pooling of samples from each location was intended to capture the overall microbial community profile representative of each major tea‐growing region in the Kangra Valley. Samples were stored at 4°C until isolation and then stored at 20°C for further studies. The soil samples were air‐dried and then analyzed for various physicochemical properties. Details of the soil physicochemical properties were provided in our previous paper on culturable diversity analysis of tea rhizosphere of the Kangra valley (Thakur et al. 2025).

### 2.2 Soil DNA Extraction and Library Preparation

DNA extracts from tea rhizosphere samples were extracted using the PowerMax Soil DNA Isolation Kit (Mo Bio Laboratories, USA), following the manufacturer's instructions. The quality of the DNA was assessed by agarose gel electrophoresis, and its concentration was measured using a NanoDrop 2000 spectrophotometer (Thermo Scientific, USA). To check bacterial and Archaeal diversity, the V3‐V4 hypervariable region of the 16S rRNA gene was amplified using primers 341f and 805r, which were coupled with Illumina Nextera adapters. Amplicons of approximately 550 bp corresponding to the target V3–V4 region were processed for library preparation. To assess fungal diversity, the ITS1 and ITS2 regions of fungal rDNA were amplified in separate PCRs using Illumina ITS1f and ITS2r primers, each appended with Illumina Nextera adapter overhangs. The resulting amplicons were visualized on an agarose gel, yielding single bands of approximately 280–350 bp for ITS1 and 320–450 bp for ITS2. The amplicons were purified using the HighPrep PCR Clean‐up System (MagBio Genomics Inc., USA). The purified product size was verified using the D1000 ScreenTape and 2200 TapeStation systems (Agilent Technologies Inc., Germany). Illumina sequencing adapters and dual indices were added using the Nextera XT Index Kit v2 (Illumina Inc., USA). The addition of Illumina adapters and indices was confirmed, and fragment size distribution was assessed using a Bioanalyzer 12000 chip on the Agilent 2100 Bioanalyzer system (Agilent Technologies, USA). The indexed libraries were then amplified using Nextera PCR Master Mix and Index 1 (i7) and Index 2 (i5) primers on a 96‐well Index Plate. Post‐amplification cleanup was carried out using AMPure XP beads (Beckman Coulter, USA). The final libraries were analyzed for fragment size and quality using the Bioanalyzer 12000 chip and 2100 Bioanalyzer system.

### 2.3 Sequencing

Sequencing of the V3‐V4 amplicon and ITS amplicon libraries was carried out on the Illumina Illumina MiSeq platform (Illumina Inc. USA). For bacterial libraries, 2 × 250 bp paired‐end sequencing chemistry was employed, while Archaeal libraries were sequenced using 2 × 265 bp paired‐end chemistry. The raw sequencing data have been submitted to the National Center for Biotechnology Information (NCBI) Sequence Read Archive (SRA).

### 2.4 Data Analysis

Raw Illumina reads were demultiplexed using the CASAVA tool (Illumina Inc. USA), and the quality of paired‐end reads was assessed using FastQC. Low‐quality reads and adapter sequences were removed during quality filtering and reads failing the predefined quality criteria were excluded from downstream analysis. Chimeric sequences were identified and removed prior to OTU clustering and taxonomic assignment. For microbial community profiling, bacterial 16S rRNA V3‐V4 and fungal ITS reads were processed using the QIIME1 pipeline. Bacterial reads were clustered into Operational Taxonomic Units (OTUs) using the UCLUST algorithm, and taxonomy was assigned based on ≥ 97% sequence similarity against the Greengenes database v13.8, a curated chimera‐free 16S rRNA reference. Archaeal 16S rRNA V3‐V4 reads were analyzed using the VSEARCH pipeline. Query sequences were aligned against the reference database using the USEARCH global method with a minimum identity threshold of ≥ 50%. Fungal ITS sequences were clustered using UCLUST against the UNITE database v7.0 and reads that did not match the reference database were subsequently clustered de novo. The sequences were taxonomically annotated using the Ribosomal Database Project (RDP) classifier, with assignments retained only when the confidence score met or exceeded a threshold of 0.5.

The statistical analysis of OTU tables generated for all samples were carried out in the R statistical platform. To understand diversity and richness of microbial communities, the alpha diversity within the samples (Richness, Shannon, Simpson and Chao) and beta diversity between the samples (Bray‐Curtis dissimilarity, Jaccard and Euclidean) were analysed. Alpha diversity indices and rarefaction curves were computed using the vegan package. Principal Component Analysis (PCA) based on diversity indices and soil properties (pH, Electrical conductivity (EC), OM, available nitrogen (N), available phosphorus (P) and available potassium (K)) was performed using the FactoMineR package (https://cran.r-project.org/web/packages/FactoMineR/index.html). For visualization of microbial abundance patterns, heatmaps were constructed using the NMF package. Venn diagrams were constructed using E Venn online tool (https://www.bic.ac.cn/EVenn/#/).

## 3 Results

**Figure 1:**
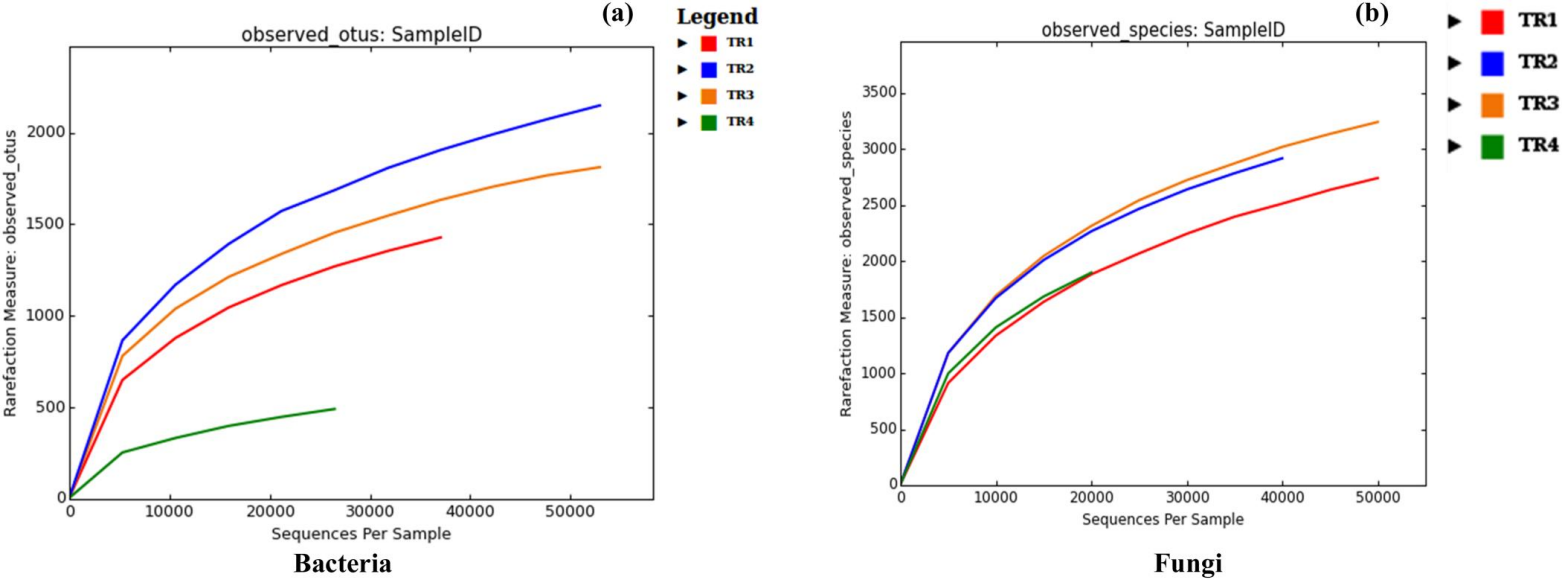
Calculated rarefaction curves of observed OTUs of (a) bacteria and (b) fungi in the 4 tea rhizosphere samples (TR1 = Dharamshala, TR2 = Baijnath TR3 = Palampur, TR4 = Joginder Nagar).

A total of 4,375,261 raw sequences were generated from 4 tea rhizosphere soil samples. Following removal and trimming, the dataset was refined to 2,614,107 reads. Among these, 1,762,878 sequences were assigned to the Domain Bacteria, 216,874 for Fungi and 634,355 for Archaea (Table 1). The rarefaction curves approached a plateau for all three microbial groups. This suggests that the observed differences in microbial richness among locations were unlikely to be substantially influenced by insufficient sequencing coverage. The rarefaction plot of the observed OTUs showed that Baijnath and Palampur samples had the highest bacterial richness, while Dharamshala and Palampur had the highest fungal richness. Samples collected from Joginder Nagar showed the lowest bacterial and fungal richness (Figure 1).

**Table 1:** Summary of read processing and taxonomic assignment.

Sample*	Total paired end reads	Processed reads	Total OTUs Picked	Total Identified rRNA sequences
Bacteria				
TR1	425,304	188,801	1440	37,994
TR2	501,699	168,552	2326	67,871
TR3	659,794	364,241	2097	86,820
TR4	1,645,282	1,041,284	514	29,769
Fungi				
TR1	174,375	80,941	3182	80,168
TR2	108,784	43,869	2986	43,194
TR3	153,642	67,854	3502	66,868
TR4	64,367	24,210	2023	23,756
Archaea				
TR1	66,229	65,919	665	65,919
TR2	189,396	187,431	950	187,431
TR3	198,560	196,066	1,022	196,066
TR4	187,829	184,939	967	184,939

^*TR1=Dharamshala, TR2=Baijnath, TR3=Palampur, TR4=Joginder Nagar.^

**Figure 2:**
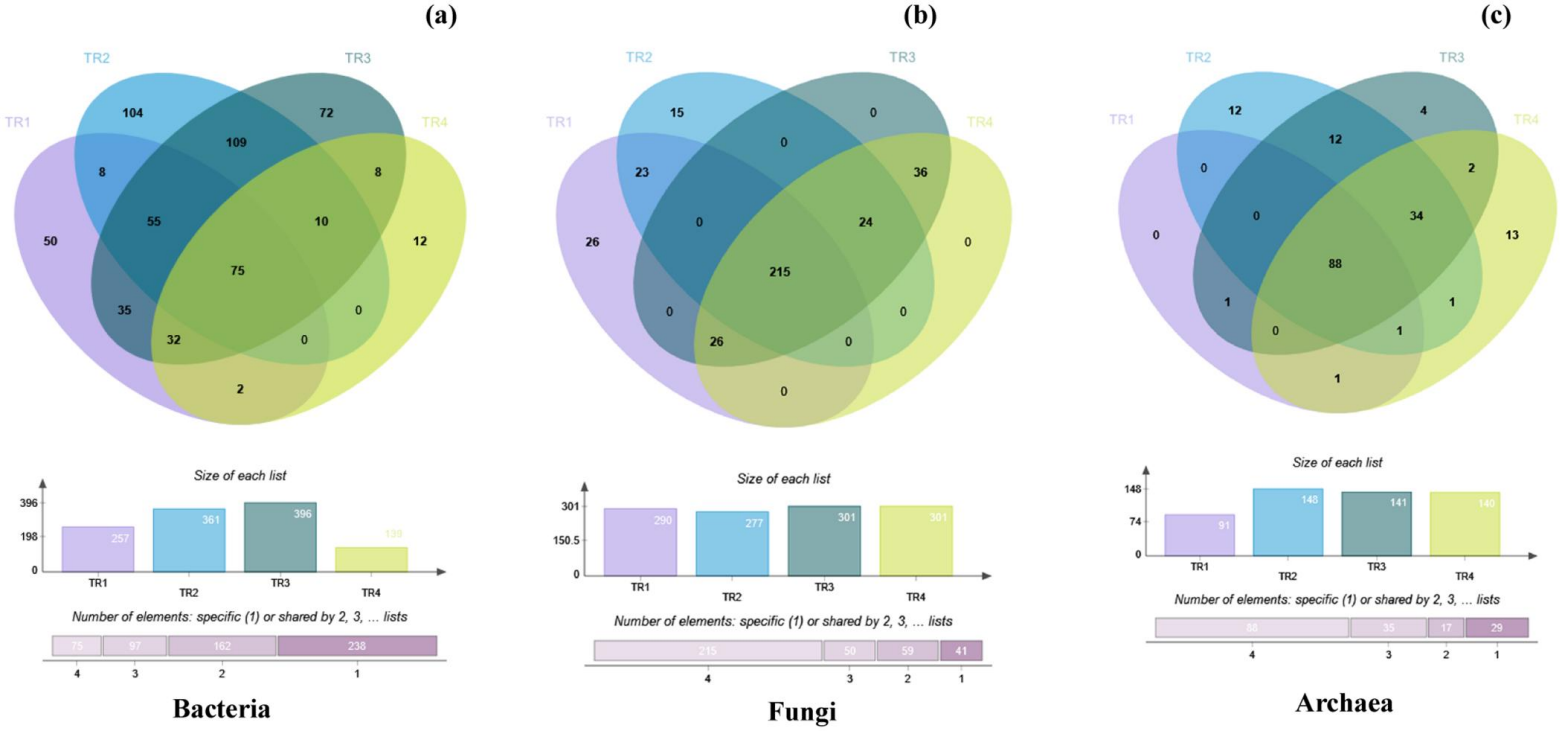
Venn diagram showing overlapping OTUs (a) bacteria (b) fungi, and (c) archaea (TR1 = Dharamshala, TR2 = Baijnath TR3 = Palampur, TR4 = Joginder Nagar).

The distribution and overlap of metagenomic elements across all the locations are shown in Figure 2. For bacterial domain, Palampur samples contained a total of 396 elements, followed by Baijnath (361), Dharamshala (257) and Joginder Nagar (139). A total of 75 OTUs are shared across all four samples, indicating the presence of a common microbiome. In addition, Dharamshala samples had 50 unique elements, Baijnath had 104, Palampur had 72, and Joginder Nagar had 12 unique elements. For the Fungi domain, samples collected from Palampur and Joginder Nagar showed the same number of metagenomic elements (301), while Dharamshala and Baijnath had a comparable number of elements. A total of 215 elements were shared across all four samples, suggesting the presence of a core microbiome. Dharamshala and Baijnath samples had 26 and 15 unique elements. For the Archaea domain, Baijnath contained a total of 148 elements, followed by Palampur (143), Joginder Nagar (140) and Dharamshala (91). Joginder Nagar also shared 34 elements with Baijnath and Palampur.

### 3.1 Abundance Analysis

**Figure 3:**
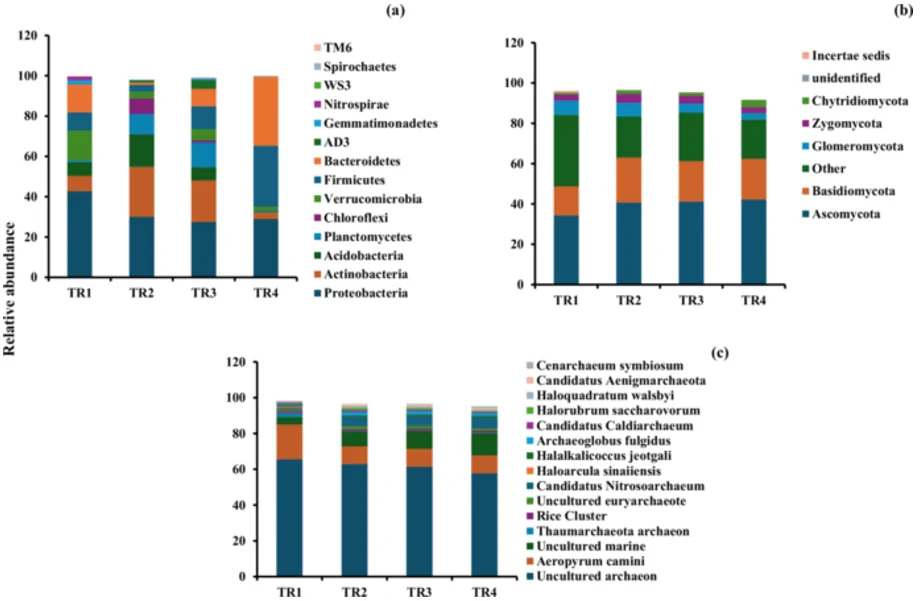
Relative abundance of microbial communities of tea rhizosphere at different locations (TR1 = Dharamshala, TR2 = Baijnath TR3 = Palampur, TR4 = Joginder Nagar). Phylum distribution of OTU showing abundance of top 10 phyla of (a) bacteria (b) fungi, and (c) archaea.

Proteobacteria were found to be the most abundant at the phylum level, accounting for 36.7%, 27.7%, 30.9% and 36.7% in Dharamshala, Baijnath, Palampur and Joginder Nagar respectively (Figure 3). Other phyla including Firmicutes (20.8%), Bacteroidetes (11.6%), Actinobacteria (6.1%), Acidobacteria (5.8%) were identified at Dharamshala; Actinobacteria (22.1%), Firmicutes (19.2%), Acidobacteria (6.3%), Chloroflexi (4.6%), Bacteroidetes (3.2%) at Baijnath; Firmicutes (22.4%), Actinobacteria (17.9%), Bacteroidetes (7.3%), Acidobacteria (4.5%), Verrucomicrobia (3.4%) at Palampur; and Firmicutes (34.9%), Bacteroidetes (7.1%), Verrucomicrobia (5.3%), Actinobacteria (4.7%), and Acidobacteria (2.9%) in Joginder Nagar soil samples. At the class level, Gammaproteobacteria, Betaproteobacteria, and Bacilli were most abundant across all the locations. At the order level, Bacillales and Burkholderiales were abundant at Dharamshala; Actinobacteria, Actinomycetales, and Bacillales were abundant in Baijnath; Actinomycetales, Bacillales, and Clostridiales were dominating at Palampur, while Bacillales, Clostridiales, and Burkholderiales were abundant in Joginder Nagar.

**Figure 4:**
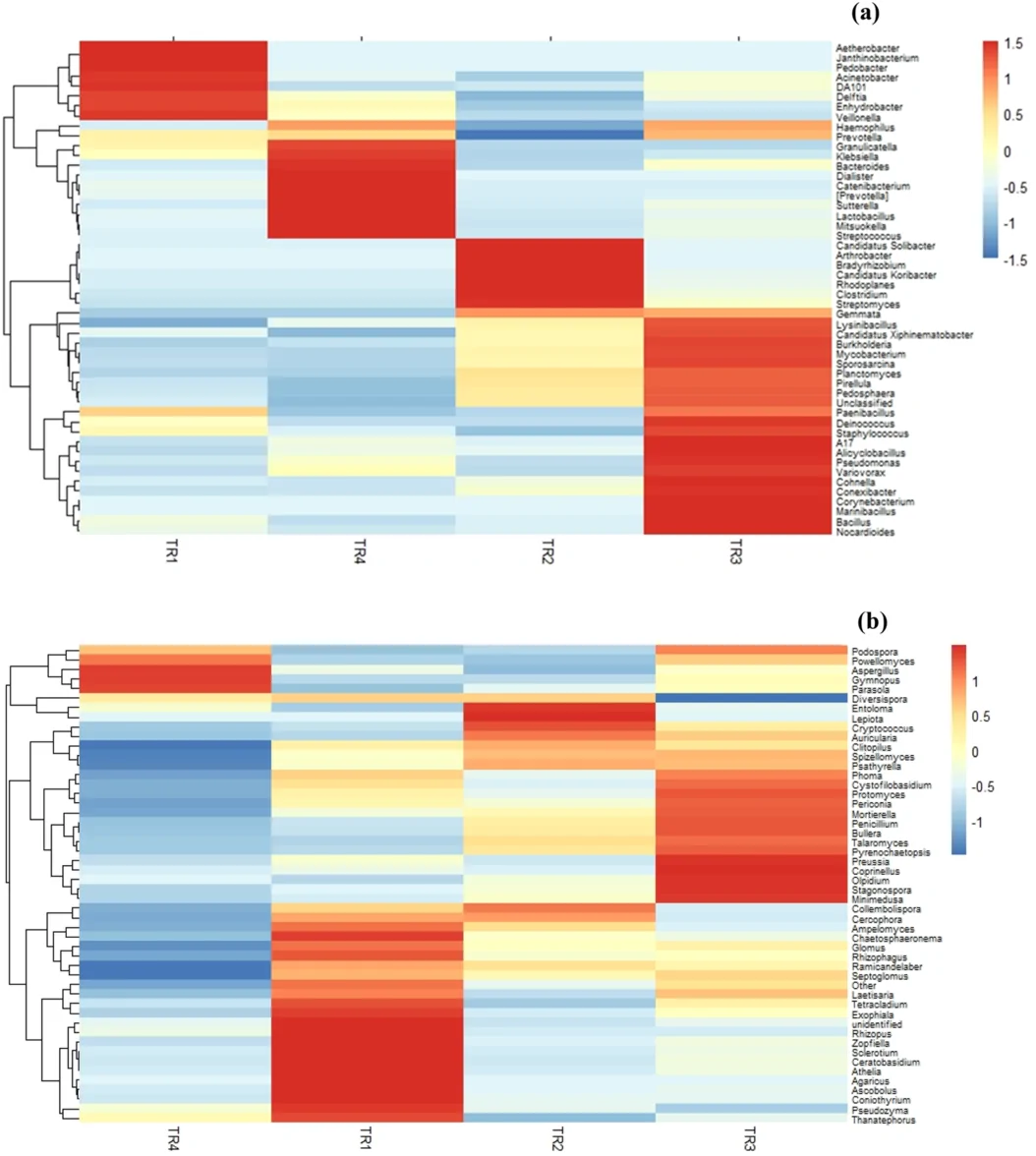
Heatmap of top 50 bacterial (a) and fungal (b) genera abundance in the 4 tea rhizosphere locations (TR1 = Dharamshala, TR2 = Baijnath TR3 = Palampur, TR4 = Joginder Nagar).

A total of 11 fungal phyla were identified among all the locations. Phyla Ascomycota, Basidiomycota, Glomeromycota, Zygomycota, and Chytridiomycota were found in all the samples. Dharamshala has a high percentage of Ascomycota, accounting for 45.6%, followed by Basidiomycota (34.2%), and Glomeromycota (8.2%). Baijnath showed the highest percentage of Ascomycota (44.5%), followed by Basidiomycota (35.0%) and Glomeromycota (7.6%). Palampur recorded the highest proportion of Ascomycota (45.8%), followed by Basidiomycota (33.5%), and Glomeromycota (7.6%). Joginder Nagar had the largest number of Ascomycota (46.2%), followed by Basidiomycota (33.3%), and Glomeromycota (8.7%). At the class level, Agaricomycetes, Dothideomycetes and Eurotiomycetes were most abundant and common among all the locations. Agaricales and Pleosporales were the most abundant fungal orders among all the locations. Palampur had recorded the highest number of genera (196), followed by Dharamshala (189), Baijnath (178), and Joginder Nagar (159). Insights of the top 50 bacterial and fungal genera is shown in Figure 4. For the Archaea domain, unidentified archaeon and *Aeropyrum camini* were the most common organisms found among all the locations. Comparative analysis of rhizobacterial species across 4 locations showed the abundance of different species. Dharamshala showed a high abundance of DA101 Unclassified (11.96%), *Prevotella copri* (8.6%) and *Janthinobacterium* unclassified (8.1%).

Alpha diversity indices displayed notable differences in the taxonomic microbial diversity (Table 2). For the bacterial domain, Palampur sample showed higher species richness (211) and a greater estimated richness (Chao1 = 241.8) but a lower Shannon index (1.75) and Simpson index (0.52), suggesting an uneven species distribution. Baijnath and Dharamshala samples also showed high species richness. In contrast, Joginder Nagar samples, although less rich (richness = 79; Chao1 = 88.7), have a higher Shannon index (2.46) and Simpson index (0.85), indicating a more even distribution of species. For Fungi, Palampur had the highest species richness (196) followed by Dharamshala (189), Baijnath (178) and Joginder Nagar (159). For archaea, Palampur showed the highest species richness (1022), followed by Joginder Nagar (967), Baijnath (950), and Dharamshala (665).

**Table 2:** Alpha diversity indices of bacteria and fungi and archaea.

Location^a^	Richness	Shannon	Simpson	Chao1
Bacteria				
TR1	131	2.11	0.74	154
TR2	170	1.79	0.66	213.2
TR3	211	1.75	0.52	241.8
TR4	79	2.46	0.85	88.7
Fungi				
TR1	189	2.28	0.71	241.8
TR2	178	2.66	0.76	203.6
TR3	196	2.57	0.75	214.7
TR4	159	2.50	0.77	177.1
Archaea				
TR1	665	3.74	0.92	913.7
TR2	950	4.26	0.96	1277.6
TR3	1022	4.32	0.96	1251.6
TR4	967	4.31	0.96	1305.3

^a^ TR1 = Dharamshala, TR2 = Baijnath, TR3 = Palampur, TR4 = Joginder Nagar.

The bacterial beta diversity indicated that samples are quite different from each other (Table 3), with high Bray‐Curtis dissimilarity values (0.4 to 0.8), high Jaccard distances (0.59 to 0.82) and high Euclidean distances (13055.8 to 42037.5). In contrast, fungal beta diversity showed a similar pattern, with moderate Bray‐Curtis dissimilarity values (0.27 to 0.59), low Jaccard distances (0.27 to 0.37) and high Euclidean distance (10613.6 to 32336.9). Archaeal beta diversity in all samples was fairly similar to each other, with low Bray‐Curtis dissimilarity values (0.12 to 0.25) and moderate Jaccard distances (0.37 to 0.44).

**Table 3:** Beta diversity indices of bacteria and fungi and archaea.

	Bray‐Curtis dissimilarity				Jaccard distance				Euclidean distance			
	TR1	TR2	TR3	TR4	TR1	TR2	TR3	TR4	TR1	TR2	TR3	TR4
Bacteria												
TR1	0	0.62	0.57	0.57	0	0.77	0.57	0.56	0	24288.4	42037.5	13055.8
TR2	0.62	0	0.42	0.81	0.77	0	0.59	0.82	24288.3	0	25483.7	33249.8
TR3	0.57	0.42	0	0.72	0.57	0.59	0	0.69	42037.5	25483.7	0	52143.0
TR4	0.57	0.81	0.73	0	0.56	0.82	0.69	0	13055.8	33249.8	52143.1	0
Fungi												
TR1	0	0.39	0.27	0.59	0	0.27	0.29	0.33	0	22364.3	11946.5	32336.9
TR2	0.39	0	0.27	0.40	0.27	0	0.28	0.33	22364.3	0	12282.9	10613.6
TR3	0.27	0.27	0	0.52	0.29	0.28	0	0.37	11946.5	12282.9	0	22540.0
TR4	0.59	0.40	0.52	0	0.33	0.33	0.37	0	32336.9	10613.6	22540.0	0
Archaea												
TR1	0	0.65	0.66	0.65	0	0.54	0.57	0.57	0	26516.8	27584.2	26711.5
TR2	0.65	0	0.12	0.25	0.54	0	0.37	0.44	26516.8	0	7374.9	14620.2
TR3	0.66	0.13	0	0.15	0.57	0.37	0	0.38	27584.2	7374.9	0	10646.7
TR4	0.6	0.25	0.15	0	0.57	0.44	0.38	0	26711.5	14620.2	10646.7	0

Abbreviations: TR1 = Dharamshala, TR2 = Baijnath, TR3 = Palampur, TR4 = Joginder Nagar.

Overall, alpha‐diversity indices showed differences in richness and evenness among the four tea‐growing locations, while beta‐diversity analysis revealed substantial differences in bacterial community composition (Bray‐Curtis dissimilarity: 0.4–0.8) but comparatively low variation among archaeal communities (0.12–0.25), indicating greater bacterial community turnover across locations.

**Figure 5:**
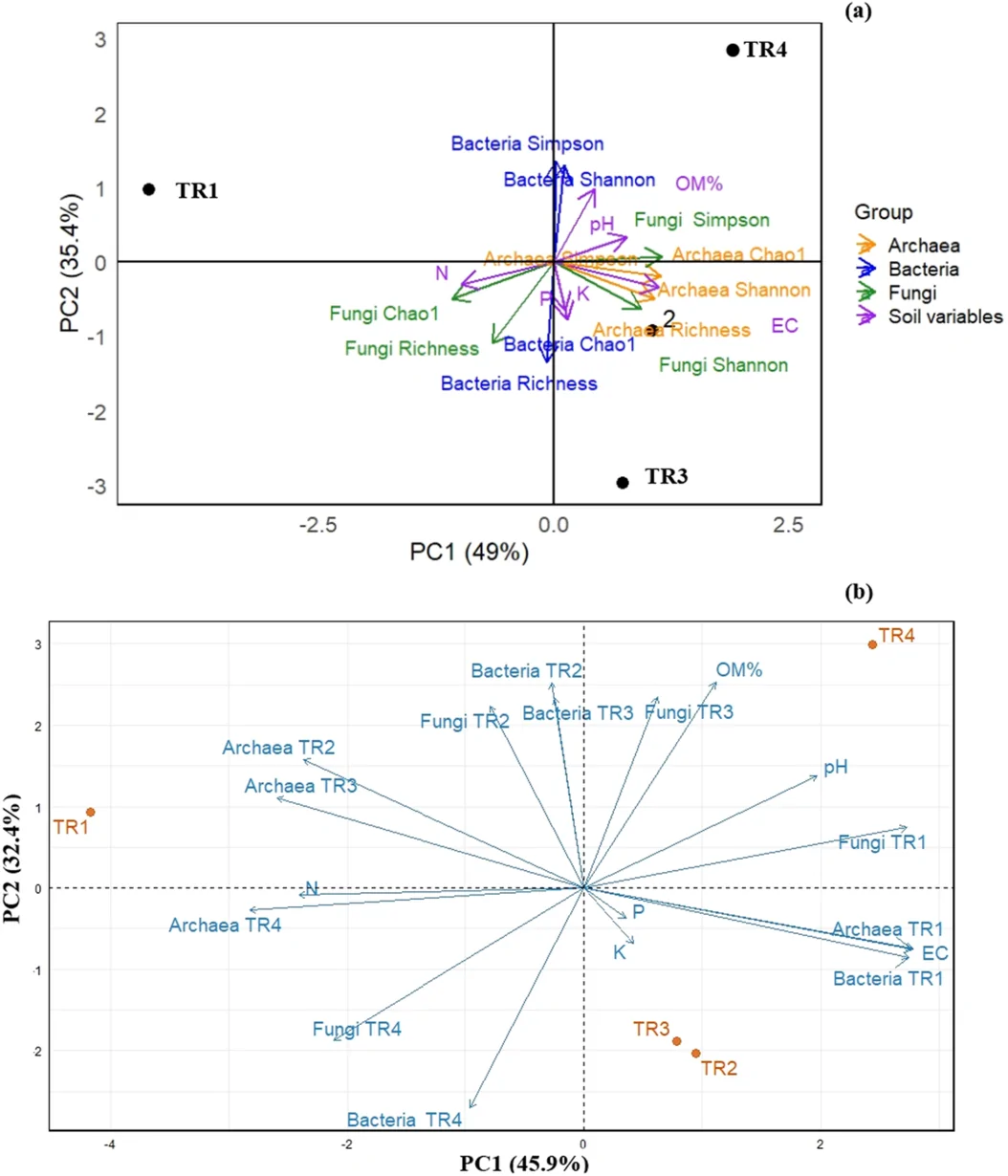
Principal component analysis based on diversity index and soil properties. (a) alpha diversity index and soil properties; (b) beta diversity index and soil properties. (TR1 = Dharamshala, TR2 = Baijnath TR3 = Palampur, TR4 = Joginder Nagar). Soil variables: EC = electric conductivity, K = potassium, N = Nitrogen, OM = organic matter, P = phosphorus.

PCA plots showed that Palampur sample had a very different bacterial community than Dharamshala, Baijnath and Joginder Nagar. Dharamshala and Joginder Nagar samples showed more similarities with each other (Supporting Information S1: Figure S2). All sampled showed variations in the fungal community. Dharamshala samples showed a very different archaeal community than the other 3 locations (Supporting Information S1: Figure S3). The first two principal components analysis of alpha diversity and soil properties explained 85% of the total variation combined (Figure 5a). PCA indicated that soil factors were variably associated with microbial diversity, with nitrogen positively aligned with fungal diversity, while EC and K were associated with archaeal diversity. pH and OM% showed moderate associations with bacterial diversity, highlighting site‐specific relationships rather than a single dominant driver. The first two principal components analysis of beta diversity and soil properties explained a combined 78% of the total variation (Figure 5b). Nitrogen was oriented towards fungal richness and Chao1, suggesting a positive association with fungal diversity. EC and K were aligned with archaeal diversity indices (Shannon and Chao1), indicating their potential influence on archaeal community structure. Soil pH and OM showed proximity to bacterial Shannon and Simpson indices, suggesting a moderate positive association with bacterial diversity. The variation in vector directions and magnitudes suggests that these relationships are site‐specific, and no single soil parameter consistently governs microbial diversity across all samples.

## 4 Discussion

This study provides insights into the microbial diversity of the tea rhizosphere of the Kangra valley. Tea rhizosphere is known for its unique environmental challenges such as poor nutrient availability, acidification, excessive use of chemical fertilizer and conventional management approaches. All these factors significantly influence the microbial community composition.

In our analysis, we found that Proteobacteria was the most abundant phylum across all the locations. Proteobacteria are the most diverse and largest phylum in terms of studied organisms. Proteobacteria have been reported in several rhizospheres, including saffron, tea, sugarcane, wheat and rice (Ambardar et al. 2021; Pivato et al. 2021; Sun et al. 2022b; Yuan et al. 2022; Zhong et al. 2025). Bacteria belonging to this phylum are known for their significance in agriculture, industrial, and medical sectors. Proteobacteria are well known to promote plant growth, biocontrol activity, and regulate the nutrient cycle in the rhizosphere. *Pseudomonas* is the most common genus of proteobacteria that has been found in many rhizospheres the and is known for its plant growth promotion and biocontrol activity (Sah et al. 2021). After Proteobacteria, Acidobacteria, Firmicutes, and Actinobacteria were the most common phyla in the tea rhizosphere. The abundance of Acidobacteria is indicative of the development of mechanisms to survive in the acidic soils due to the use of pesticides and fertilizers over time. A similar pattern of phyla Proteobacteria, Actinobacteria, and Acidobacteria was found in Darjeeling tea rhizosphere (Bhattacharyya et al. 2022). Several other studies on tea rhizosphere metagenomic analysis conducted in China have also shown the dominance of Proteobacteria and Acidobacteria during short‐term and long‐term tea plantations (Arafat et al. 2020; Li et al. 2016).

The predominance of taxa such as Proteobacteria, Actinobacteria, Acidobacteria and Firmicutes in the tea rhizosphere is ecologically relevant, as these groups are associated with nutrient cycling, organic matter decomposition and adaptation to the acidic soil conditions characteristic of tea cultivation (Chopra et al. 2020). In particular, genera such as *Bacillus* and *Pseudomonas* are recognised as important plant‐growth‐promoting rhizobacteria with potential roles in nutrient solubilisation, phytohormone production, siderophore production and biological control of soil‐borne pathogens (Bhattacharyya et al. 2022). Their occurrence in the tea rhizosphere may therefore contribute to plant growth and soil health, although their specific functional roles in the Kangra Valley require further experimental validation.

In our previous study on tea culturable rhizobacteria, *Bacillus* was found dominant, followed by *Pseudomonas* and *Burkholderia* (Thakur et al. 2025). *Bacillus* species are generally known to dominate the tea rhizosphere (Pandey and Palni 1997). Dominance and abundance of microbial diversity can be influenced by a wide range of factors such as environmental, meteorological, temporal and seasonal (Peng et al. 2023). Seasonal variations such as summer, winter, or monsoon significantly impact microbial community composition and diversity (Jia et al. 2020). Thus, the timing of sampling must be cautiously documented as it can significantly influence the microbial diversity data interpretation.

Fungi are key drivers in soil ecological functions, and a wide range of fungal diversity is an indicator of soil quality (Frąc et al. 2018). Fungi are well‐known biocontrol agents and play an important role in suppressing plant pathogens in the soil (Thakur et al. 2024a). Dominance of species of *Penicillium* and *Trichoderma* was found in the tea rhizosphere of the Kangra valley. The dominance of certain fungal species and the suppression of other species is likely due to antagonistic effect of the other fungal or bacterial species. In addition, the climate of the location in this study, which is a subtropical climate (Sharma and Chandel 2023) with significant monsoon influence, could also shape the abundance of the Fungi. Other abiotic factors, such as soil pH, salinity, and temperature factors could also influence the fungal diversity in the rhizosphere. In this study phyla Ascomycota and Basidiomycota dominated in all the locations. Phylum Ascomycota has potential for abiotic stress tolerance and biocontrol activity due to the production of secondary metabolites is perhaps the reason for its abundance (Challacombe et al. 2019). High abundance of phyla Ascomycota and Basidiomycota was also observed from the rhizospheres of native plants in Atacama Desert, *Solanum tuberosum*, *Atractylodes macrocephala* and *Panax notoginseng* ((Fuentes et al. 2020; Qin et al. 2017; Tan et al. 2017; Zhou et al. 2025). Tea rhizospheres are well documented for the diverse presence of arbuscular mycorrhizal fungi (AMF), which form a symbiotic relationship with the tea plant (Bag et al. 2022; Zhang et al. 2022). Some Proteobacteria belonging to the genera *Burkholderia*, *Enterobacter*, *Klebsiella*, *Pseudomonas*, and *Rhizobium* have been reported to enhance AMF functions of plant growth promotion and biocontrol activity in the rhizosphere (Nasslahsen et al. 2022).

Several studies have shown that the soil properties and environmental conditions within the rhizosphere can influence the richness and evenness (Deng et al. 2024; Lamb et al. 2011). Soil nutrients such as N, P, and K are critical for microbial growth and proliferation. Nitrogen is essential for plant growth and development, and plant assimilates the nitrogen from the soil as nitrite, nitrate, or ammonia. Tea cultivation is heavily dependent on the chemical fertilizers as nutrient availability is low in acidic soils (Zayed et al. 2023). Little is known about the long‐term effects of fertilization on tea rhizosphere microbial diversity. We found the available N was significantly associated with the microbial diversity. A study conducted in China showed that nitrogen addition substantially altered the microbial composition in the N‐limited soils (Zhou et al. 2022). In contrary, N addition negatively affected soil microbial diversity and reduce the relative abundance of Actinobacteria and Nitrospirae (Wang et al. 2018). Our study showed a negative association with soil pH, OM and EC and microbial diversity. This suggests possible sensitivity of fungal communities to salinity or soil ionic content. In contrary, a Chinese study found that changes in EC and other soil properties regulate the fungal diversity (Yang and Sun 2020). However, it is challenging to draw definitive conclusions about what particular soil variable influences the microbial diversity. Soil physicochemical properties, particularly EC, pH, organic matter, and nutrient availability were key drivers shaping microbial community structure across treatments. Distinct clustering of treatments indicates differential microbial responses, with bacteria and Fungi associated with nutrient‐rich conditions, while Archaea were linked to comparatively nutrient‐limited environments. Further longitudinal and multi‐seasonal studies are required for a better understanding that can account for spatial and temporal variability.

The observed associations between soil properties and microbial diversity suggest that factors such as pH, OM, nitrogen, EC and K may contribute to differences in microbial community structure across the tea rhizosphere. Previous studies have similarly identified soil pH and nutrient availability as important factors influencing tea‐associated microbial communities, with pH reported as a key driver of bacterial and fungal community composition (Wei et al. 2023). However, these relationships should be interpreted cautiously because correlations do not establish causation, and microbial communities may simultaneously be influenced by tea cultivar, elevation, climate, soil management and other environmental factors (Zhang et al. 2022). The relatively high bacterial dissimilarity and distinct clustering of Palampur and Dharamshala further suggest that local environmental conditions may strongly influence rhizosphere community structure. However, these potential drivers should be interpreted cautiously because each location was represented by a single composite sample and therefore within‐site variability could not be assessed. Archaeal communities showed relatively similar composition across the four locations, as indicated by the low Bray‐Curtis dissimilarity (0.12–0.25), although Palampur and Dharamshala showed differences in archaeal richness and community structure. The predominance of unidentified archaea and *Aeropyrum camini* suggests that archaeal communities may contribute to key soil biogeochemical processes, including carbon and nutrient cycling. However, functional roles cannot be confirmed from the present taxonomic data alone and require further functional investigation.

### 4.1 Limitations

Although the present study revealed substantial variation in bacterial, Fungal, and Archaeal community composition, the functional roles of the identified microbial communities remain speculative because functional genes and metabolic pathways were not directly assessed. Future studies incorporating shotgun metagenomic, metatranscriptomic or other functional approaches are needed to validate the predicted functions and establish their ecological significance in the tea rhizosphere.

## 5 Conclusion

These findings provide a baseline understanding of the tea rhizosphere microbial diversity in the Kangra Valley and may support the development of microbiome‐based strategies for sustainable tea cultivation. In particular, the identification of dominant and location‐specific microbial taxa provides a foundation for future studies investigating beneficial microorganisms for improving soil health, nutrient availability, plant growth, and resilience to environmental stresses. This study complements our previous culture‐based investigation by providing a molecular characterisation of the tea rhizosphere bacterial community, together offering a more comprehensive understanding of its microbial diversity. Functional metagenomics can also be helpful in predicting the associations between microbes and tea plant metabolism under various stressful environmental conditions in the changing climate.

## Author Contributions

**Rishu Thakur:** conceptualization, writing – original draft, data curation, investigation. **Hena Dhar:** data curation, writing – review and editing. **Shashi Kiran:** data curation, writing – review and editing. **Arvind Gulati:** conceptualization, writing – review and editing, resources, supervision.

## Funding

The authors have nothing to report.

## Ethics Statement

The authors have nothing to report.

## Consent

The authors have nothing to report.

## Conflicts of Interest

The authors declare no conflicts of interest.

## Supporting information


Supporting File

## Data Availability

The data that support the findings of this study are openly available in National Centre for Biotechnology Information (NCBI) at https://www.ncbi.nlm.nih.gov/, reference number Sequence Read Archive (SRA). (SAMN56668015, SAMN56668016, SA.
