# Supplementary material for: Metagenomic Insights Into Microbial Diversity of Tea Rhizosphere of the Kangra Valley
Source: Microbiologyopen. 2026 Sep 9;15(5):e70416. doi: 10.1002/mbo3.70416 (PMC13555615; doi:10.1002/mbo3.70416)
Supplement: Supplementary file 1 [file MBO3-15-e70416-s001.docx]

**Supplementary Fig S1:** Sample sites

**(a)**

**(b)**

**(c)**

**Supplementary Figure S2:** PCA analysis based on bacterial (a), fungal (b) and archaeal (c) genera of tea rhizosphere of the Kangra Valley.

**Supplementary Figure S3:** Correlation analysis between alpha diversity indices and soil properties of tea rhizosphere of the Kangra Valley.
